# Dissecting the clinical heterogeneity of early-onset Alzheimer’s disease

**DOI:** 10.1038/s41380-022-01531-9

**Published:** 2022-04-07

**Authors:** Daniel W. Sirkis, Luke W. Bonham, Taylor P. Johnson, Renaud La Joie, Jennifer S. Yokoyama

**Affiliations:** 1 Memory and Aging Center, Department of Neurology, Weill Institute for Neurosciences, University of California, San Francisco, San Francisco, CA 94158 USA; 2 Department of Radiology and Biomedical Imaging, University of California, San Francisco, San Francisco, CA 94158 USA

**Keywords:** Diseases, Genetics, Neuroscience

## Abstract

Early-onset Alzheimer’s disease (EOAD) is a rare but particularly devastating form of AD. Though notable for its high degree of clinical heterogeneity, EOAD is defined by the same neuropathological hallmarks underlying the more common, late-onset form of AD. In this review, we describe the various clinical syndromes associated with EOAD, including the typical amnestic phenotype as well as atypical variants affecting visuospatial, language, executive, behavioral, and motor functions. We go on to highlight advances in fluid biomarker research and describe how molecular, structural, and functional neuroimaging can be used not only to improve EOAD diagnostic acumen but also enhance our understanding of fundamental pathobiological changes occurring years (and even decades) before the onset of symptoms. In addition, we discuss genetic variation underlying EOAD, including pathogenic variants responsible for the well-known mendelian forms of EOAD as well as variants that may increase risk for the much more common forms of EOAD that are either considered to be sporadic or lack a clear autosomal-dominant inheritance pattern. Intriguingly, specific pathogenic variants in *PRNP* and *MAPT*—genes which are more commonly associated with other neurodegenerative diseases—may provide unexpectedly important insights into the formation of AD tau pathology. Genetic analysis of the atypical clinical syndromes associated with EOAD will continue to be challenging given their rarity, but integration of fluid biomarker data, multimodal imaging, and various ‘omics techniques and their application to the study of large, multicenter cohorts will enable future discoveries of fundamental mechanisms underlying the development of EOAD and its varied clinical presentations.

## Introduction

**Fig. 1:**
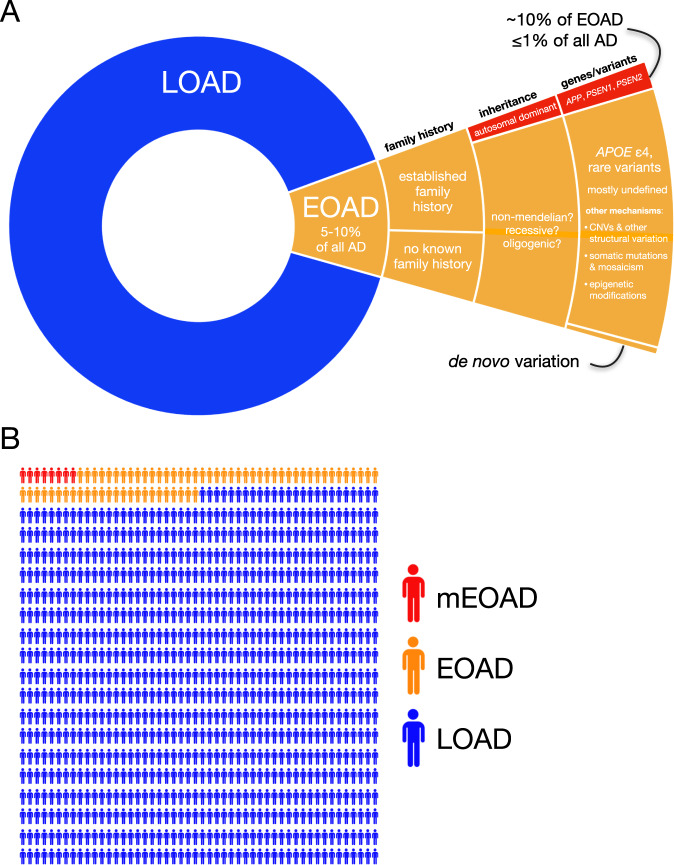
The prevalence of early-onset Alzheimer’s disease. **A** A hierarchical pie chart illustrates the prevalence of EOAD in relation to LOAD. EOAD is thought to represent ~5–10% of all AD [1]. While only ~10% of EOAD cases are thought to be due to autosomal-dominant inheritance [3, 4, 11], a substantial proportion (perhaps a majority) of EOAD cases have a positive family history [19]. The major genes implicated in mEOAD are *APP*, *PSEN1*, and *PSEN2*, while the primary risk factor for non-mendelian EOAD is the *APOE* ε4 allele [4]. Rare variants in ~20 additional genes have been implicated in risk for EOAD and its clinical variants. In addition, a small number of apparently sporadic EOAD cases with an unusually young age of onset have been shown to harbor de novo *PSEN1* variants [186]. Given the estimated ~90–100% heritability for EOAD [11], additional variants mediating EOAD risk are likely to be discovered. Additional genetic mechanisms likely to be studied with increasing intensity in the coming years include copy number variants (CNVs) and other structural variation, somatic variation and mosaicism, and epigenetic modifications. **B** In a random sample of 1000 individuals with AD, we might expect ~50–100 people to have EOAD; fewer than 10 would be expected to have mEOAD.

Alzheimer’s disease (AD), the most common cause of dementia, is an insidious neurodegenerative disease that affects memory or other cognitive functions and is characterized by the accumulation of amyloid-β (Aβ) peptides and hyperphosphorylated tau protein in the brain. AD is currently estimated to affect more than six million people in the United States, and the number of affected individuals is expected to more than double by 2050 [1]. Most patients with AD are diagnosed after age 65, and more than 10% of all individuals in this age group are currently living with AD. Many additional people, perhaps five million or more, are thought to have mild cognitive impairment (MCI) due to neuropathological changes characteristic of AD [1]. In contrast to the more common, late-onset form of AD (LOAD) described above, an estimated 5–10% of individuals with AD (corresponding to ~300,000–700,000 people in the US, based on the current prevalence of AD [1]) develop symptoms before age 65 (Fig. 1; [2–4]). Although these individuals with early-onset AD (EOAD) make up a small fraction of all AD cases, they are more likely to experience an aggressive clinical course [5, 6]; more likely to display an atypical clinical presentation (e.g., involving visuospatial, language, executive, or motor dysfunction) [7–9]; take longer on average to obtain a correct diagnosis [9]; may experience unique social and financial disruptions because of their relatively young age [5, 6]; and are often excluded from participation in clinical trials [10]. In this review, we will (i) provide an overview of EOAD and its heterogeneous clinical manifestations, including the more prevalent atypical forms of disease; (ii) summarize the latest advances in fluid biomarker and molecular neuroimaging research; (iii) describe how structural and functional imaging can inform EOAD diagnosis and translational research; (iv) assess the genetic variation associated with EOAD risk and the molecular pathways such variation implicates; and (v) discuss how this information has deepened our understanding of the potential pathogenic mechanisms underlying EOAD, its clinical variants, and AD as a whole.

The age cutoff distinguishing EOAD from LOAD is, by definition, an arbitrary one. Although most studies use a cutoff of 65 years, some instead use a cutoff of 60 (e.g., [11]; reviewed in [4]). EOAD and LOAD are both defined by the same neuropathological changes—Aβ peptides that accumulate extracellularly as amyloid plaques and hyperphosphorylated tau protein that accumulates intraneuronally as neurofibrillary tangles (NFTs)—but pathological changes in EOAD are less likely to include co-occurring TDP-43 pathology, hippocampal sclerosis, or vascular injury [4, 12], and this is likely a reflection of the younger age of onset. Consistent with this notion, co-occurring TDP-43 pathology appears to be similarly infrequent in mendelian and non-mendelian EOAD [12, 13]. On the other hand, individuals with EOAD may have more severe [14] and pervasive tau neuropathology relative to LOAD, as indicated by higher tau-PET signals [15–17] and more severe atrophy [18] relative to LOAD.

A common misconception regarding EOAD is that the term is synonymous with mendelian forms of AD, which most often present at a young age. In fact, mendelian EOAD (mEOAD; sometimes referred to as familial EOAD), represents only a small minority of EOAD cases (Fig. 1). Nearly all described cases of mEOAD involve autosomal-dominant inheritance and may account for ~10% of all EOAD ([3, 4, 11]). The vast majority of mEOAD cases in which a pathogenic variant has been determined are caused by variants in the amyloid precursor protein (*APP*) or the presenilin genes (*PSEN1*, *PSEN2*) encoding the catalytic subunit of the ɣ-secretase complex that generates Aβ peptides via cleavage of APP. Interestingly, it has been estimated that about one-quarter [19] (or perhaps an even higher proportion [20]) of mEOAD families have as-yet uncharacterized variants, indicating that causal variants in additional genes may yet be discovered [21]. Given that EOAD has an estimated heritability of ~90–100%, additional genetic variants contributing risk in the large proportion of EOAD cases that are non-mendelian are also likely to be discovered. Indeed, common and rare variants in >20 genes, as well as trisomy 21 [22], are known or suspected to increase risk for EOAD. In contrast to EOAD, LOAD is a highly polygenic disease likely to involve thousands of variants, with all but a handful of such variants individually contributing a very small amount of risk for AD [11, 23]; reviewed in [24]. On the other hand, polygenic models do not fit the inheritance pattern observed in EOAD [11], and genetic risk for EOAD is therefore likely to be mediated by a smaller number of variants in a recessive or oligogenic framework.

Recent years have witnessed dramatic improvements in our ability to conceptualize AD within a molecular framework, aided primarily by advances in fluid biomarker research, neuroimaging, and genetics. Throughout this review, we endeavor to describe the state of the art for each area of research, with the goal of showing how the described methods and approaches can be used in concert to generate new discoveries; improve our understanding of—and diagnostic acumen for—EOAD and its heterogeneous clinical presentations; and address the key questions of how EOAD arises and how it differs from LOAD.

## Clinical and neuropsychological features of eoad

Patients with EOAD can present with a variety of clinical signs, symptoms, and syndromes that do not always resemble the typical amnestic syndrome most often described in LOAD. EOAD is associated with less salient memory deficits and greater likelihood of impairment of other functions, including language, visuospatial, executive, and motor functions, and behavioral dysregulation. This clinical heterogeneity is partly responsible for the misdiagnosis and delay in diagnosis for patients with EOAD [25]. However, non-amnestic (i.e., atypical) presentations of AD have gained better recognition in the last decade—while “progressive memory worsening” was one of the mandatory features of “probable AD” in the 1984 clinical criteria [26], this requirement was dropped in the 2011 revision of the diagnostic guidelines [27].

The heterogeneity of clinical presentations of EOAD can be seen as a spectrum based on patient’s relative impairment in various cognitive and clinical domains. Some patients at extreme ends of this spectrum fulfill criteria for specific non-amnestic syndromes which are also referred to as focal cortical presentations [28] or atypical phenotypes [9]. These presentations are associated with insidious onset (typically before age 65) and gradual progression of clinical deficits.

The most thoroughly described non-amnestic phenotype is posterior cortical atrophy (PCA; [29]), which is characterized by difficulties in space and object perception; Balint syndrome (simultanagnosia, optic ataxia, oculomotor apraxia); Gerstmann syndrome (dyscalculia, dysgraphia, left-right confusion, finger agnosia); constructional, dressing, or limb apraxia; environmental agnosia; and alexia, with the relative sparing of other functions [30]. While most cases with PCA have underlying AD neuropathology, other neurodegenerative etiologies are possible, including Lewy body disease, corticobasal degeneration, and prion disease [30].

The logopenic variant of primary progressive aphasia (lvPPA; [31]) is the language presentation of AD, with predominant impairment of single-word retrieval in spontaneous speech and naming, difficulty repeating phrases and sentences, and phonological errors. In contrast, single-word comprehension, object knowledge, and motor speech are preserved and agrammatism is absent.

Other presentations of AD have been described, mainly involving executive function and behavior [32]. These phenotypes—which are sometimes referred to as frontal AD [33]—are challenging to diagnose because their presentation overlaps with the behavioral variant of frontotemporal dementia (bvFTD) [34, 35]; efforts are ongoing to better refine the clinical features that characterize frontal AD and distinguish it from bvFTD [36, 37]. In EOAD, executive dysfunction usually involves deficits in working memory, cognitive flexibility and set shifting, leading to difficulty in planning and multitasking. Behavior-predominant presentations of AD are relatively rare and their symptoms overlap with bvFTD, although the behavioral variant of AD usually features less disinhibition, compulsiveness, or hyperorality and more neuropsychiatric symptoms (e.g., agitation, delusions, hallucinations) than bvFTD.

Patients with AD can also present with corticobasal syndrome (CBS), characterized by predominant motor and sensory symptoms (including limb rigidity, bradykinesia, dystonia, myoclonus, apraxia, alien limb phenomenon). Unlike PCA and lvPPA, CBS is not highly predictive of underlying AD neuropathology—less than a third of patients with CBS have AD [28, 38, 39]; the majority have underlying frontotemporal lobar degeneration (FTLD), most often the corticobasal degeneration subtype. Without biomarkers, antemortem prediction of the neuropathological diagnosis remains difficult in CBS [38, 40].

In addition to a high degree of variability in the domains of cognitive impairment, EOAD is usually associated with a more aggressive clinical course than LOAD [41] as cognitive and clinical function tends to decline more rapidly in younger patients [12, 42–45].

While the above-mentioned syndromes are mostly described in non-mendelian EOAD, clinical presentations are heterogeneous in mEOAD as well, with variable patterns of cognitive and behavioral impairment. In addition, mEOAD is associated with a higher prevalence of non-cognitive neurological symptoms such as myoclonus, seizures, spastic paraparesis, and extrapyramidal signs [46–48].

## Fluid biomarkers

Fluid biomarkers provide a snapshot of soluble molecular components related to pathophysiologic processes and can inform disease diagnosis and/or prognosis. Measurements of Aβ, total tau (t-tau), and phosphorylated tau (p-tau) in the cerebrospinal fluid (CSF) and, increasingly, plasma represent key biomarkers that can accurately differentiate AD from other neurodegenerative diseases as well as healthy controls. Due to the clinical heterogeneity of EOAD, additional fluid biomarkers with high sensitivity and specificity may be needed to increase the precision with which we monitor and understand EOAD and its clinical variants.

### Latest advances in Alzheimer’s disease fluid biomarkers

The past several years have witnessed dramatic advances in the analysis of p-tau species in the CSF and plasma as potential AD biomarkers (reviewed in [49, 50]). Measurements of plasma tau phosphorylated at residues 181 and 217 (p-tau181 and p-tau217) in particular have shown that these tau species have remarkable accuracy as candidate biomarkers of AD diagnosis and progression, both in CSF [51–55] and plasma [56–67]. However, much work remains to be done in determining the validity of these biomarkers in diverse populations [68] that have thus far been underrepresented in these studies.

### Lessons from mendelian early-onset Alzheimer’s disease

Studies of mEOAD families, particularly from the Dominantly Inherited Alzheimer Network (DIAN), have provided important insights into early biomarker changes that are also relevant for more common forms of AD. Seminal work on mEOAD families enrolled in DIAN reported reductions in CSF Aβ_42_ (which is inversely related to accumulation of intracerebral, fibrillar Aβ) among pathogenic variant carriers that could be detected as early as 25 years before the expected age of onset (interpolated from cross-sectional data), with significant differences reported between carriers and non-carriers 10 years before expected onset [69]. In the same study, CSF t-tau was found to be significantly increased relative to non-carriers 15 years prior to expected symptom onset [69]. More recently, longitudinal analyses of mEOAD participants have refined our understanding of the temporal ordering of fluid biomarker changes, confirming significantly higher CSF p-tau181 levels in carriers compared to non-carriers in baseline measurements, but also finding longitudinal declines in p-tau181 among carriers starting ~5 years before expected symptom onset [70]. P-tau181 and p-tau217 have both been found to be elevated in CSF ~20 years prior to expected symptom onset; p-tau217 was also shown in the same study to be a highly sensitive marker for identifying individuals who are Aβ-positive by PET imaging (AUC of 0.97; [51]). Along similar lines, plasma p-tau217 is significantly elevated in an independent kindred of *PSEN1* p.E280A carriers relative to non-carriers starting ~20 years before expected symptom onset [57], a finding that is strikingly consistent with the results from DIAN [51]. If these biomarker dynamics are conserved in non-mendelian EOAD, one would expect to see elevated p-tau217 in plasma as early as age 40 in an individual who will go on to develop AD symptoms at age 60. Thus, for individuals with a family history of early-onset dementia (but who lack known pathogenic variants), such biomarker screening could eventually become quite useful, particularly once more-effective therapies become available.

### Fluid biomarkers in early- vs. late-onset Alzheimer’s disease

In contrast with mEOAD and LOAD, relatively few studies have assessed fluid biomarker levels in non-mendelian EOAD. However, a recent meta-analysis suggests that changes in CSF Aβ_42_, t-tau, and p-tau are generally consistent between mendelian and non-mendelian forms of EOAD, with the possible exception that t-tau may show a greater elevation—relative to controls—in symptomatic mEOAD compared to non-mendelian EOAD [71]. On the other hand, an early study directly comparing EOAD to LOAD found that CSF Aβ_42_ was significantly lower in EOAD [72], consistent with the notion of greater pathological burden in EOAD relative to LOAD [14]. It is not currently known if levels of plasma p-tau181 or p-tau217 are increased to a greater extent in EOAD relative to LOAD.

### Fluid biomarkers in atypical clinical variants

Few studies have directly compared fluid biomarkers in typical vs. atypical clinical variants of EOAD. Moreover, the reported fluid biomarker results for PCA have thus far been inconsistent, with variable results reported by different groups [67, 73–76]. With regard to CBS, careful examination of data published last year reveals that the subset of patients with CBS who are Aβ-positive by PET imaging—and therefore presumably have CBS due to AD neuropathology—have clearly elevated levels of p-tau181 and p-tau217 relative to individuals with CBS who are Aβ-negative [67]. Taken together, these studies suggest that atypical clinical syndromes of EOAD including lvPPA and CBS generally have fluid biomarker changes that are similar to those observed in amnestic EOAD (and AD generally), while PCA requires further study.

## Molecular imaging in eoad

The development of radiotracers binding to fibrillar Aβ deposits in the mid 2000s (Pittsburg compound B, Florbetapir, Florbetaben, Flutemetamol, Flutafuranol [77]) and to tau-containing paired helical filaments in the 2010s (including Flortaucipir, MK-6420, PI-2620, RO-948 [78]) has dramatically changed the field by allowing researchers and clinicians to detect and track the development of the neuropathological hallmarks of AD in living patients. More recently, new tracers have been developed to monitor pathophysiological changes beyond amyloid and tau (e.g., synaptic density [79], neuroinflammation [80], mitochondrial metabolism [81], cholinergic denervation [82]), but we will not describe these studies in the present review due to the paucity of data in EOAD.

### Amyloid-PET

**Fig. 2:**
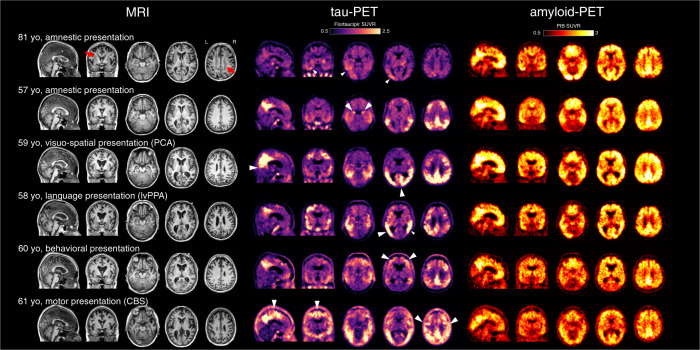
Structural MRI, tau-PET, and amyloid-PET in representative cases with LOAD and EOAD subtypes. Patients with typical amnestic LOAD (top row) show brain atrophy with white matter lesions (red arrows), mild to moderate tau-PET signal predominantly in the temporal and parietal lobes (white arrowheads), and global cortical amyloid-PET signal. Additional clinical subtypes of EOAD are illustrated (rows 2–6). Tau-PET signal is greater in EOAD compared to LOAD and the regional distribution mirrors the clinical syndromes; white arrowheads indicate phenotype-specific features. For example, medial temporal binding is observed in amnestic variant, parieto-occipital binding in visuospatial variant (PCA), a left-predominant pattern in language variant (lvPPA), higher frontal binding in behavioral presentations, and high perirolandic binding in motor presentations (CBS). Atrophy can be observed on the MRI in regions with high tau-PET signal. Amyloid-PET does not show robust association with age of onset or clinical features. Images courtesy of Gil Rabinovici, UCSF Memory and Aging Center.

Amyloid-PET imaging is most often interpreted dichotomously (negative or positive) based on the absence or presence of diffuse cortical tracer binding [83]. Multiple PET-to-autopsy studies have shown that amyloid-PET is a reliable marker of fibrillar β-amyloid pathology [84–86], although the earliest stages of amyloidosis (Thal stages 1–2 [87]) might not be detected [88, 89]. Unlike most neuroimaging modalities, the regional distribution of amyloid-PET signal is usually non-informative—PET signal is typically seen throughout the neocortex, with little difference observed across clinical phenotypes [90–93] (Fig. 2). In studies including patients with sporadic AD (EOAD and LOAD), amyloid PET signal shows no clear relationship with age of onset or clinical severity [90, 91].

In mEOAD, atypical amyloid-PET patterns have been reported in some cases, such as high striatal [94–96] or cerebellar [97] binding; the latter finding has to be considered when quantifying amyloid-PET signal given that the cerebellum is often considered pathology-free and used as a reference region [98]. Moreover, amyloid-PET lacks the sensitivity to detect Aβ in specific genetic variants that result in atypical Aβ neuropathology with low neuritic plaque burden, the type of fibrillar deposits to which PET tracers mainly bind [99]. For instance, *PSEN1* carriers usually show a high frequency of diffuse, cotton wool Aβ deposits that are associated with a lower PET signal than that observed for typical neuritic plaques [100]. Specific *APP* variants (Arctic *APP* and E693Δ) have been associated with low fibrillar Aβ burden, resulting in negative amyloid-PET scans [101–103]. More broadly, an extensive investigation of 206 individuals with mEOAD from the DIAN study recently emphasized the impact of various pathogenic variants in *APP*, *PSEN1*, and *PSEN2* on the temporal dynamics and regional patterns of amyloid-PET [104].

### Amyloid-positivity in asymptomatic individuals

Data from cohorts of patients with mEOAD have shown that amyloid-PET signal starts to emerge 15–25 years prior to expected symptom onset [69, 70, 105]; similar timing has been observed in non-mendelian AD [106, 107]. Altogether, converging evidence supports a long asymptomatic (or preclinical) phase [108] in both mendelian and non-mendelian AD. This dissociation or time lag between amyloid-PET positivity and clinical deficits requires caution when interpreting amyloid-PET scans in patients evaluated for dementia. Indeed, the frequency of incidental amyloid-PET-positivity in cognitively normal individuals increases with age (<1% below age 55 vs. 35% around age 80), rendering a positive amyloid-PET scan relatively uninformative in older patients. In contrast, positive amyloid scans are very informative in non-mendelian EOAD given the low frequency of incidental positive scans at a younger age.

### Tau-PET

Radiotracers that bind to AD tau pathology have shown great promise for improving patient diagnosis, prognostication, and clinical trial design. Tau-PET is elevated in the cortex of patients who are positive for amyloid biomarkers and allows differentiation of patients with AD from controls or patients with other neurodegenerative diseases with high accuracy [109]. PET-to-autopsy studies [89, 110, 111] have shown that tau-PET can reliably detect advanced levels of tau pathology that are believed to account for cognitive impairment and dementia (Braak stages IV–VI). Further research is needed to determine whether earlier stages of tau pathology can be detected with PET.

### Regional distribution matters

In contrast to amyloid imaging, tau-PET provides information that correlates with clinical features. Although most patients with AD show tau-PET signal in the medial and inferior temporal cortex, regional patterns are highly variable across patients and tau-PET usually mirrors both the regional patterns of neurodegeneration (i.e., brain atrophy observed on MRI) and clinical symptoms. Patients with PCA have high tau-PET signal in the occipital and parietal cortex, while patients with lvPPA have a higher burden in the left temporo-parietal areas, and patients with amnestic AD have relatively higher medial temporal lobe tau-PET signal (see Fig. 2 and [90, 93, 112, 113]). Similarly, patients with CBS due to AD neuropathology have tau-PET signal in perirolandic areas [114–116]. Above and beyond differences between these atypical phenotypes, tau-PET correlates with cognitive deficits in a regionally-specific manner [117, 118].

More recent data shows that tau-PET could help forecast future cognitive decline [119] better than amyloid-PET or MRI-derived measures. Patterns of baseline tau-PET can also predict severity and regional distribution of future brain atrophy at the individual patient level, especially in patients with EOAD [120]. Altogether, tau-PET has strong potential as a precision medicine biomarker and has been integrated into clinical trials to select patients more likely to respond to anti-amyloid therapies [121].

### Tau and age of onset

Converging evidence has shown that patients with non-mendelian EOAD have higher tau-PET signal than patients with LOAD, especially in frontal and parietal lobes [15, 16, 122]. This phenomenon is consistent with post mortem findings [12] and cannot be explained by the overrepresentation of atypical, non-amnestic presentations in groups of patients with EOAD—the negative association between tau burden and age of onset exists within groups of patients with amnestic EOAD as well as PCA or lvPPA [90, 91]. Remarkably, this higher tau burden is not accompanied by more severe clinical or cognitive symptoms at the time of PET: patients with EOAD thus seem to be able to tolerate a higher pathology burden than patients with LOAD. This observation might be related to higher cognitive resilience (i.e., younger patients might be able to cope with higher levels of pathology, for instance through more efficient functional compensation mechanisms) [123], or because the higher level of co-existing neuropathologies in LOAD lowers the amount of tau pathology required for symptom manifestation [124, 125]. To this day, little data is available regarding tau-PET in mEOAD, and while all studies show that tau-PET elevation occurs later than amyloid-PET changes, the precise timing of tau-PET changes during the presymptomatic phase is still unclear [126–128].

## Structural and functional neuroimaging

Given the distinct clinical phenotypes observed in EOAD, it comes as no surprise that there are similarly distinct neuroanatomic signatures localizing to brain regions involved in each clinical syndrome’s affected cognitive domains (Fig. 2). Moreover, patterns of regional cortical atrophy are tightly linked to the distribution of tau neuropathology [90, 92, 93, 112, 113]. Neuroimaging thus facilitates systematic characterization of each EOAD clinical variant’s distinct radiographic signature, monitoring of disease progression, and quantitative integration of brain structure and function with other biomarkers of disease.

### Mendelian EOAD: a window into structural changes in presymptomatic EOAD

Neuroimaging is a valuable lens through which the field can view both nascent and established mEOAD, offering unique insights into the pathobiology and progression of EOAD and perhaps, by extension, LOAD. Indeed, genetic and molecular analyses of mEOAD families led to the discovery of *APP* and the early formulations of the ‘amyloid cascade’ hypothesis that has driven decades of research and drug development in LOAD [129]. However, it should be noted that considerable uncertainty remains regarding the extent to which neuroimaging abnormalities in mEOAD can be extrapolated to either non-mendelian EOAD or LOAD. The use of neuroimaging in the study of mEOAD dates back at least 20 years [130]. Since then, multiple large mEOAD cohorts have been assembled—the largest and most-established of which is the multinational DIAN cohort [131]. The foundational work of the DIAN study and other similar cohorts is reviewed elsewhere [132] and we thus focus the remainder of this section on more recent developments.

The past several years are defined by expanded use of multimodal imaging and the introduction of advanced machine learning algorithms to study prodromal mEOAD. Recent work from DIAN that combined diffusion tensor imaging (DTI) with functional imaging demonstrated that white matter changes in the forceps major and minor (key tracts linking cortical hubs in the default mode network [DMN], a group of functionally linked brain regions most impacted in AD) predate symptom onset by up to 10 years [133]. In a recently published manuscript with implications for both EOAD and normal aging, Gonneaud et al. elegantly paired advanced machine learning methods with functional imaging to predict chronologic age across the adult lifespan and subsequently showed that presymptomatic mEOAD cases demonstrate an MRI-predicted brain age greater than their chronologic age [134]. Building on these findings, Franzmeier et al. integrated multimodal imaging (structural MRI, FDG-PET, and amyloid PET) with CSF biomarkers (Aβ_42_, p-tau181, and t-tau) using neural networks to predict cognitive decline in mEOAD with replication in a LOAD cohort [135]. Using this model’s results to risk-stratify a simulated intervention, the authors estimated that use of this framework would enable sample size reductions of up to 50–75% [135]. Additional research will be required to test whether these models can be adapted to other forms EOAD, though they provide a valuable framework for future studies.

### Amnestic EOAD and LOAD

While some studies of smaller-sized cohorts report no morphologic differences between amnestic EOAD and LOAD [136], the preponderance of evidence points toward more pronounced global cortical atrophy in EOAD and focal involvement of cortex in the medial temporal, occipital, dorsal frontal, and parietal regions (Fig. 2; [137–140]). This stands in contrast to LOAD, which predominantly involves the medial temporal cortex (Fig. 2; [138, 140]). The underlying reasons for these morphological differences remain an active of area of research, but dysregulation of multiple interconnected brain networks may play an important role. Evidence from functional neuroimaging studies suggests that amnestic EOAD’s distinct anatomy may be related to disruption of additional brain networks beyond the DMN, including those implicated in executive control [141–143]. In addition, DTI highlights white matter differences between amnestic EOAD and LOAD, with the former demonstrating decreased white matter integrity (fractional anisotropy) in the inferior fronto-occipital fasciculus as well as the anterior and posterior cingulum [144]—key tracts linking brain regions and cortical networks critical for executive function [145]. In contrast, clinically evident white matter disease (as measured by white matter hyperintensities and/or lacunes) is known to be enriched in LOAD relative to EOAD [146, 147]. In this regard, the aforementioned fractional anisotropy findings suggest that white matter disease may play a distinct and underappreciated role in EOAD and that a more nuanced approach to evaluating white matter changes is indicated in future studies. Taken together, these findings provide a compelling argument for the morphologic uniqueness of amnestic EOAD relative to LOAD.

### Non-amnestic clinical variants

Beyond the well-established gray matter atrophy patterns observed in lvPPA [148–150] and PCA [150–152], recent literature continues to highlight neuroimaging as a vital tool in the study of atypical EOAD. For example, recent work shows that hippocampal subfield volumes vary uniquely between amnestic EOAD and PCA despite both regions demonstrating atrophy [153]. On the other hand, a study from the same year found whole hippocampal volumes did not effectively differentiate typical from atypical EOAD [154], suggesting that more detailed evaluation of brain structure may differentiate additional EOAD subtypes. These findings build on prior multimodal imaging analyses which illustrated unique changes in gray matter atrophy [150], white matter integrity [155], and functional connectivity [150, 156] corresponding to each syndrome’s unique neuroanatomic distribution superimposed upon a shared background of neurodegeneration in regions resembling the distribution of the DMN.

### The future of structural neuroimaging

Neuroimaging alone provides useful information about disease state and progression, but its value is enhanced when paired with other biomarkers of disease. In this context, neuroimaging may not only elucidate the pathophysiology of EOAD but also provide prognostic data useful in clinical trials and patient care. Work in non-mendelian EOAD that integrates imaging and CSF biomarkers has shown promising results, but a limitation of these studies has been their small sample sizes and emphasis on amnestic EOAD [139, 157]. These limitations will be addressed by upcoming studies of multicenter cohorts such as the Longitudinal Early-Onset Alzheimer’s Disease Study (LEADS), which is actively collecting clinical, genetic, CSF, and neuroimaging data [10]. Using this data for analyses structured similarly to the mEOAD work described above may help to disentangle the complex genetic and pathophysiologic underpinnings of the clinical heterogeneity seen in EOAD.

## The genetics of early-onset Alzheimer’s disease

### Identification of the major mendelian early-onset Alzheimer’s disease genes

A crucial genetic discovery in EOAD—which has been massively influential for the course of research into all forms of AD—occurred 30 years ago with the mapping of a missense variant in *APP* that segregated with disease in an autosomal-dominant EOAD family [158]. Subsequent studies identified pathogenic variants in *PSEN1* and *PSEN2* in additional autosomal-dominant AD families [159–161]. Analysis of LOAD families has more recently resulted in the identification of rare, risk-conferring variants and established pathogenic variants in *APP*, *PSEN1*, and *PSEN2*, thus suggesting that these genes may also be relevant for the more common, late-onset variety of AD [162, 163]. Additional studies using data from the AD Sequencing Project also provide suggestive evidence that rare variation in *PSEN1* increases risk for LOAD [164, 165]. Given the potent influence that the amyloid cascade hypothesis [166, 167] has had on the field and on AD drug development, the finding that variants in these genes may also confer risk for LOAD provides important support for the generalizability of this hypothesis (now updated [168, 169]) to all forms of AD.

### *APOE* and risk for typical early-onset Alzheimer’s disease

Apolipoprotein E (*APOE*) is the major genetic risk factor for LOAD, with the common ε4 allele increasing risk for AD by threefold in heterozygotes and 12-fold in homozygotes [170–172]; reviewed in [173]. Although the ε4 allele was initially associated with LOAD, given that it is known to reduce the age at onset (AAO) [170], it is not surprising that this variant also increases risk for EOAD [174, 175]. Intriguingly, among individuals with EOAD, the ε4 allele seems to contribute risk specifically for classical, amnestic EOAD—patients with atypical, non-amnestic presentations are less likely to be ε4 carriers [176–178]. The ε4 allele’s association with medial temporal lobe pathology that is characteristic of amnestic AD may explain its weaker association with atypical forms of EOAD, which show clinical syndrome-specific regional atrophy patterns beyond the medial temporal lobe. Beyond the risk imparted by *APOE* ε4, rare variation in another apolipoprotein gene, *APOB*, may also increase risk for EOAD [179].

It remains unclear precisely how *APOE*-mediated risk for AD is related at a biochemical level to the pathologic cascade driven by disease-causing variants in *APP*, *PSEN1*, and *PSEN2*. However, a rare patient that belongs to a large Colombian mEOAD kindred of *PSEN1* p.E280A carriers—who showed symptom onset nearly 30 years later than expected—may hold important insights. This patient was found to be a homozygous carrier of the rare *APOE* Christchurch variant (p.R136S; [180]), which may exert its protective effect by breaking the biochemical link between increased Aβ and the initiation of tau pathology [180, 181].

### Genetic risk for atypical forms of Alzheimer’s disease

Comparatively little information exists regarding genetic risk for atypical forms of AD beyond that discussed above for *APOE* ε4. However, one genome-wide association study (GWAS) for PCA risk identified significant association with the *APOE* locus but found a smaller odds ratio than is seen for typical AD [182], consistent with the studies cited above. Three additional loci (*SEMA3C*, *CNTNAP5*, and *FAM46A*) in this study reached genome-wide significance and represent promising candidates for replication [182]. Although lvPPA is most often associated with AD pathology (reviewed in [9]), lvPPA due to non-AD pathology also occurs, and these cases are often associated with *GRN* variants (see below for further discussion of *GRN*) [183].

### Age-at-onset variation

Variation in the AAO of MCI and/or dementia occurs in mEOAD and is known to be highly heritable [184, 185]. Such variation can occur both as a function of the particular pathogenic variant [186] and within large families harboring a given pathogenic variant. For example, whole-genome sequencing in members of a very large, extended kindred harboring the *PSEN1* p.E280A pathogenic variant has revealed a common haplotype in a chemokine gene cluster—which includes a missense variant within *CCL11*—that associates with markedly later (~10 years) disease onset [185]. In the same study, a coding variant in *IL4R*, encoding the interleukin 4 receptor, was also suggested as a potential modifier of AAO in p.E280A carriers. A separate study of families harboring the *PSEN1* p.G206A pathogenic variant has identified additional loci that may modify AAO in EOAD and potentially LOAD [187]. More work is needed in this important area, including systematic investigations such as the ones highlighted here, in additional mEOAD families.

### Limitations and interpretation of genetic studies of early-onset Alzheimer’s disease

In the sections that follow, we highlight associations of several genes (e.g., *MAPT*, *PRNP*, *GRN*) with clinical EOAD that are better known for their role in other neurodegenerative diseases. When interpreting the results of genetic studies of EOAD, it is important to ask several questions. First, is the phenotype in question clinically diagnosed EOAD or pathologically confirmed EOAD? If the former, is there good reason to believe the underlying neuropathology is AD? (For example, is amyloid- or tau-PET data provided? Are p-tau181 or p-tau217 levels known and are they consistent with AD?) If such evidence is not presented, interpretation of the results should be shaped by the possibility that any identified variants may actually contribute to clinical EOAD via non-AD pathology. The importance of these questions is further highlighted by the broad spectrum of clinical EOAD—and particularly the atypical syndromes—which may overlap with that of etiologically distinct neurodegenerative disorders. Finally, it is important to remain cognizant of the fact that many genetic studies have focused, for reasons of cost and practicality, on genes already known to be implicated in AD and other forms of dementia or neurodegeneration. The above factors and biases have cumulatively played an important role in shaping our current knowledge regarding the genetics of EOAD.

### *MAPT* variation in clinical early-onset Alzheimer’s disease

**Fig. 3:**
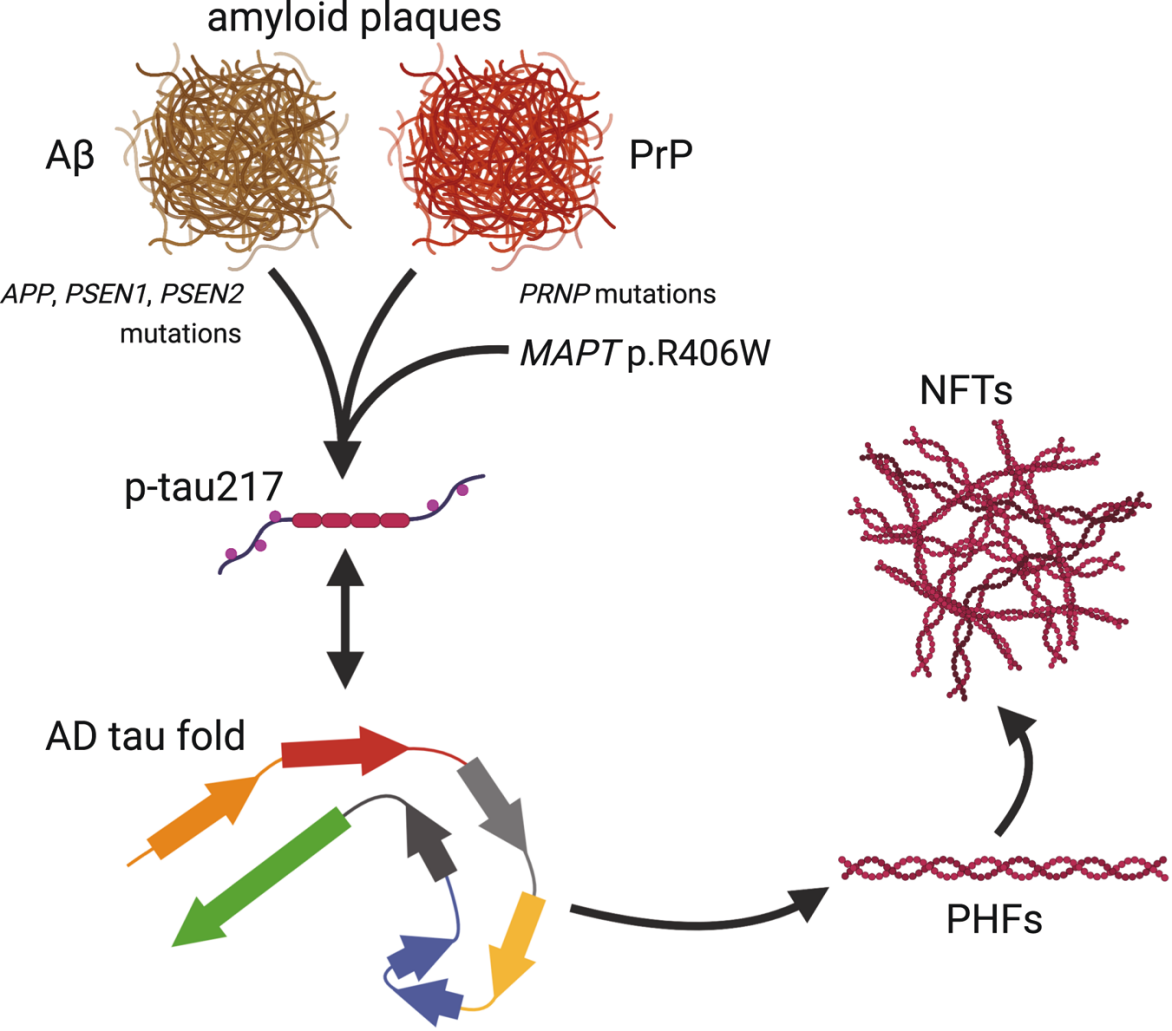
Alzheimer’s disease tau pathology can be induced by distinct amyloids and specific *MAPT* variants. The AD tau fold is normally thought to occur downstream of Aβ fibril or plaque formation. However, neuropathological analyses of rare cases with pathogenic *PRNP* variants and prion disease suggest that amyloids composed of prion protein (rather than Aβ) may also be sufficient to induce the tau fold characteristic of AD [199], phosphorylation of tau at threonine 217 (p-tau217; [199]), and the formation of PHFs and NFTs [197, 199]. While extracellular amyloid is generally assumed to be required for the initiation of this pathogenic cascade, rare individuals harboring the *MAPT* p.R406W variant and presenting with clinical AD suggest that this variant may circumvent the requirement for upstream amyloid [192, 193] and therefore may represent an alternative starting point for the production of p-tau217 [193] and generation of PHFs [188, 192] and NFTs [192] characteristic of AD. It is currently unclear precisely how p-tau217 is related to the formation of PHFs and NFTs. The AD tau fold drawn here is represented as in Hallinan et al., 2021.

A subset of variants in *MAPT*—encoding the microtubule-associated protein tau and the causative gene for chromosome 17-linked familial FTD with parkinsonism [188]—have been found in cases of early-onset dementia resembling clinical AD. In particular, the p.R406W variant is often associated with a clinical phenotype resembling EOAD [189–192]. This phenotype may be connected to the observations that p.R406W tau can form the paired helical filaments that make up NFTs in AD [188] and that individuals harboring the p.R406W variant have abnormal levels of p-tau217—a marker that is otherwise very specific for AD—in the absence of amyloid pathology (Fig. 3; [193]). Independent of causative alleles, the rare p.A152T variant of *MAPT* has been identified in several individuals with sporadic EOAD [194] after having previously been found to increase risk for both AD and FTD [195].

### *PRNP*: an alternative trigger for tau pathology in early-onset Alzheimer’s disease?

Homozygosity at codon 129 of *PRNP*—encoding the prion protein associated with Creutzfeldt-Jakob disease—has been associated with increased risk for EOAD in both sporadic cases and those with a family history [196]. An additional rare PRNP variant (p.Q160X) has been found in a family that had an AD-like clinical presentation with severe NFT pathology, prion protein deposits, and a lack of Aβ-positive plaques [197] and in additional patients with clinical EOAD [198]. Although it remains unclear how the prion protein is connected to tau pathology, work from last year suggests that the pathological tau species associated with the p.Q160X and p.F198S variants of *PRNP* bears striking resemblance to that found in AD (i.e., NFTs composed of paired helical filaments), lending support for the notion that extracellular amyloid aggregates—whether composed of Aβ or prion protein—may represent a general trigger for the formation of tau NFTs [199] and downstream neurodegeneration (Fig. 3).

### *GRN*: risk factor for clinical early-onset Alzheimer’s disease?

Potentially pathogenic variants in several genes more commonly associated with FTLD, including *GRN* (encoding progranulin) and *C9orf72*, have also been associated in rare cases with clinical EOAD [200, 201]. Strikingly, *GRN* variants have also been identified in LOAD families at a frequency comparable to that of variants in the canonical mEOAD genes *APP*, *PSEN1*, and *PSEN2* [162]. Although these *GRN* + LOAD cases were noted to be clinically indistinguishable from other cases of probable AD, the authors speculated that autopsy of such patients would likely reveal FTLD rather than AD pathology [162]. In addition, a large-scale whole-exome sequencing study has identified suggestive rare variant enrichment in *GRN* in LOAD [202]; interestingly, one such patient had definite AD pathology, indicating that *GRN* variation associated with clinical AD may not always be due to underlying FTLD pathology [202]. An additional report is consistent with this possibility, although the presence of the *APOE* ε4 allele in such *GRN* variant carriers complicates the interpretation [203]. In addition, a significant variant near *GRN* was identified in a very large LOAD GWAS last year [204], although some caution is warranted in the interpretation of this finding given the use of proxy dementia phenotype data in this study. Taken together with the significant association of a variant in *TMEM106B*—a well-known risk modifier for FTLD with TDP-43 pathology [205–207]—with LOAD in the same study, the data suggest that *GRN* and *TMEM106B* may be involved in risk for multiple, apparently disparate forms of neurodegeneration. Although it is unclear how such risk would be imparted mechanistically, the shared role of *GRN* and *TMEM106B* in maintaining white-matter homeostasis represents a plausible mechanism (reviewed in [208–210]).

### Additional genes implicated in early-onset AD: *SORL1*, *ABCA7*, *TREM2*, *TYROBP*, and others

**Fig. 4:**
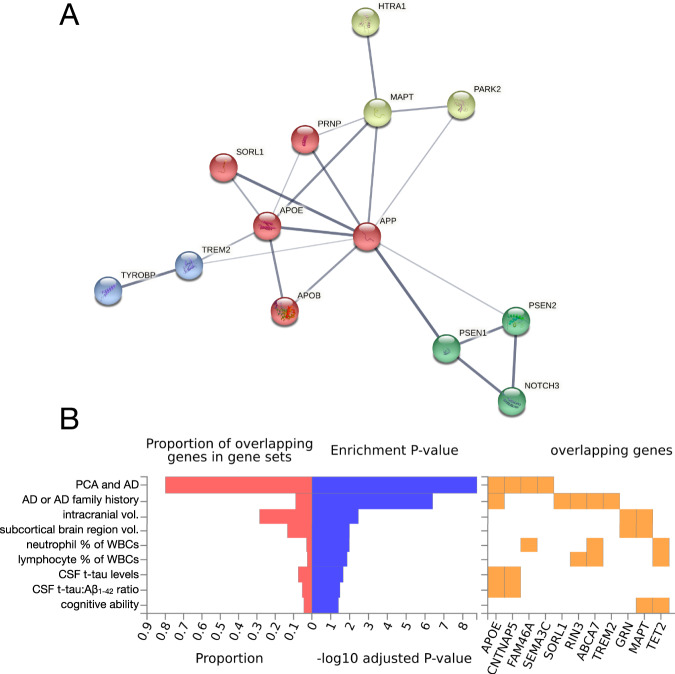
Interaction network and GWAS enrichment analyses of early-onset Alzheimer’s disease-associated genes. **A** A list of 26 EOAD- and PCA-associated genes^1^ was submitted for analysis via the STRING database (v 11.5; [238]) to visualize potential physical and functional interactions between the encoded proteins. The analysis recapitulated well-known interactions while also revealing an interaction between HTRA1 and MAPT, reflecting HTRA1’s ability to degrade aggregated and fibrillar tau [239, 240]. Further review of the literature reveals that HTRA1 is capable of degrading APP and APOE in addition to tau [241, 242]. This suggests that rare, deleterious variation within *HTRA1* might increase EOAD risk (as suggested in [225]) not only via mechanisms related to CARASIL/CADASIL (i.e., in a manner analogous to *NOTCH3* pathogenic variants), but also potentially via effects on tau, APP, or APOE metabolism. Querying the full STRING network with this gene set and limiting active interaction sources to experiments and databases, we obtained a protein–protein interaction enrichment *p* value of 1.1 × 10^−16^. Network edge thickness indicates the strength of the supporting data; nodes are colored according to cluster identity resulting from MCL clustering. **B** The same gene set was submitted to the FUMA GWAS platform (*GENE2FUNC* function; [243]) to determine whether EOAD- and PCA-associated genes were enriched in sets of significantly associated genes for a large number of GWAS phenotypes. The analysis revealed significant (*p*_FDR_ < 0.05) enrichment for expected phenotypes (e.g., AD and family history of AD, PCA, CSF t-tau levels), but also revealed significant enrichments for phenotypes like intracranial volume, subcortical brain region volumes, relative neutrophil and lymphocyte abundance, and cognitive ability. A subset of the significant FUMA GWAS results were selected for display. ^1^Gene list: *APP*, *PSEN1*, *PSEN2*, *APOE*, *APOB*, *SEMA3C*, *CNTNAP5*, *FAM46A*, *CCL11*, *MAPT*, *PRNP*, *GRN*, *C9orf72*, *SORL1*, *ABCA7*, *TREM2*, *TYROBP*, *PSD2*, *TCIRG1*, *RIN3*, *RUFY1*, *NOTCH3*, *HTRA1*, *CHCHD10*, *PARK2*, and *TET2* (all references provided within the main text).

Due to space limitations, we summarize additional genes implicated in EOAD risk in Table 1. These include *SORL1* [202, 211–215], reviewed in [173]; *ABCA7* [216–218], reviewed in [219]; *TREM2* [220, 221]; *TYROBP* [222, 223]; *PSD2* [3]; *NOTCH3* [224]; *HTRA1* [225]; *CHCHD10* [225], reviewed in [208]; *PARK2* [194]; and several others. To explore the relationships between these genes, we performed a network analysis using the STRING database, which highlighted an intriguing, putative mechanistic connection between HTRA1 and EOAD risk (Fig. 4A) involving not only tau but also amyloid and APOE. Moreover, we performed functional annotation of established and suspected EOAD risk genes described within this review using the FUMA GWAS platform (Fig. 4B) and found significant associations with phenotypes such as intracranial and subcortical brain region volumes, relative abundance of multiple classes of leukocytes, as well as cognitive ability. The insights provided by these tools and analyses may help contextualize novel EOAD genetic risk factors identified in the future.

**Table 1:** Additional genes associated with early-onset Alzheimer’s disease (EOAD) risk or harboring rare, potentially deleterious variants in EOAD.

*Gene*	*Chr. location*	*Protein*	*Year associated with EOAD*	*Odds ratio*	*Reference*
*APOE*	19q13.32	Apolipoprotein E	1994	3^a^; 12^b^	van Duijn et al.
*SORL1*	11q24.1	Sortilin-related receptor	2012	12^c^	Pottier et al.
*NOTCH3*	19p13.12	Neurogenic locus notch homolog protein 3	2012	NR	Guerreiro et al.
*TREM2*	6p21.1	Triggering receptor expressed on myeloid cells 2	2013	4	Pottier et al.
*ABCA7*	19p13.3	Phospholipid-transporting ATPase ABCA7	2016	3	Le Guennec et al.
*TYROBP*	19q13.12	TYRO protein tyrosine kinase-binding protein	2016	NR	Pottier et al.
*PSD2*	5q31.2	PH and SEC7 domain-containing protein 2	2017	NR	Kunkle et al.
*TCIRG1*	11q13.2	V-type proton ATPase 116 kDa subunit A3	2017	2^d^	Kunkle et al.
*RIN3*	14q32.12	Ras and Rab interactor 3	2017	5^d^	Kunkle et al.
*RUFY1*	5q35.3	RUN and FYVE domain-containing protein 1	2017	19^d^	Kunkle et al.
*PARK2*	6q26	E3 ubiquitin-protein ligase parkin	2017	NR	Barber et al.
*HTRA1*	10q26.13	Serine protease HTRA1	2021	NR	Jiao et al.
*CHCHD10*	22q11.23	Coiled-coil-helix-coiled-coil-helix domain-containing protein 10, mitochondrial	2021	NR	Jiao et al.

These genes, most of which were not discussed in the main text due to space constraints, have been associated with EOAD through a combination of rare variant enrichment analyses, family studies, or the identification of putatively damaging variants in genes previously implicated in other neurodegenerative or neurological disorders. *APOE* is included in the table as a point of reference.

^a^Approximate odds ratio for a single copy of the *APOE* ε4 allele.

^b^Approximate odds ratio for two copies of the ε4 allele.

^c^Approximate odds ratio for very rare *SORL1* variants predicted to be highly damaging.

^d^These genes showed suggestive associations with EOAD. NR, odds ratio not reported. Additional references for these genes are given in the main text.

### Additional mechanisms: de novo variants, copy number variation, epigenetic modifications, and somatic variation

In individuals with apparently sporadic EOAD with very early-onset (below age 51), variants in *PSEN1* were identified in 13% of cases in one study, and in the subset of cases for which parental DNA was available, all variants were found to have occurred de novo [186]. These findings suggest that *PSEN1* may be an important contributor to apparently sporadic AD cases with very early-onset (≤50 years), either due to de novo variants or because the case’s transmitting parent died before the onset of AD (thus precluding a positive family history). Besides smaller variants in *MAPT*, a rare duplication encompassing the *MAPT* locus is known to underlie some cases of early-onset dementia resembling AD clinically [226]. Additional copy number variations (CNVs) and other structural variation have also been found in mEOAD, including duplication of *APP* [227], deletion of *PSEN1* exon 9 [228, 229], and other rare CNVs involving additional genes [230, 231]. In addition, work from our group has implicated rare variation in *TET2*—encoding an enzyme that promotes DNA demethylation—in EOAD as well as FTD risk [232]. Given that methylation within *SORL1*, *ABCA7*, and other loci has been associated with AD risk [233, 234], the results suggest that epigenetic modifications (and genetic variation that affects such modifications) should be explored further for their contribution to EOAD risk. Finally, further exploration of mosaicism [235] and brain somatic variation for their role in AD (reviewed in [236]) represent a promising area for future research.

## Conclusions

Recent advances in our understanding of the multifaceted clinical, molecular, and genetic underpinnings of EOAD have highlighted the complexity and nuances of how amyloid and tau interact with brain structure and function to produce a strikingly heterogenous array of EOAD phenotypes which variably overlap with LOAD. Within this framework, emerging work within mEOAD offers a unique opportunity to understand the subtle CSF, PET, and MRI biomarker changes that occur decades prior to symptom onset. Genetic analyses in non-mendelian clinical EOAD suggest that the pathophysiology of EOAD (and by extension LOAD) is more complex than previously thought and may, in very rare cases, occur independently of Aβ neuropathology.

While the multidisciplinary advances featured above represent the culmination of decades of painstaking clinical, genetic, and molecular research, many open questions remain and are ripe for addressing in future work. A few examples are as follows:

First, given that EOAD is estimated to be ~90–100% heritable and only ~10% of cases are attributable to mendelian variants, where does the remaining ~80–90% of heritable risk lie across the human genome? A portion of this risk is hypothesized to be due to recessive variation, but direct evidence for this has remained largely elusive (with some exceptions, e.g., [237]). Additional areas that require deeper scientific exploration include rare variation, methylation changes, the cumulative effect of multiple common and/or rare variants (i.e., oligogenic risk), and the factors mediating AAO variation.

Second, what are the genetic and pathophysiologic underpinnings of the marked phenotypic heterogeneity observed in EOAD? Given the common endpoint of Aβ plaques and tau NFTs, there is strikingly little molecular and even less genetic data to explain why some EOAD patients develop syndromes such as lvPPA, frontal AD, or CBS, while others develop the more frequently observed amnestic phenotype.

Third, as multimodal MRI and PET biomarkers of AD become more sensitive and specific to disease status and progression, what role will CSF and plasma biomarkers such as Aβ and p-tau play in clinical research and trials? In the era of amyloid- and tau-PET scans and the burgeoning utility of plasma biomarkers, the clinical necessity of lumbar puncture may eventually be limited to specific scenarios in which particular analytes are required. Moreover, plasma biomarkers are expected to become even more useful globally because many clinical research centers that may not have PET imaging capabilities will nevertheless be able to analyze plasma biomarkers. Clearly, many fundamental questions remain regarding EOAD etiology and clinical heterogeneity, but we now have a powerful array of tools in place to robustly tackle these questions in the coming years. Despite the emergence of novel technologies and modes of analysis, it is important to remain mindful that most neuroimaging, fluid biomarker, and genetic analyses conducted to date have been performed in affluent and well-educated individuals of European ancestry and are thus not representative of the global community. This limitation represents an important problem that needs to be addressed in future studies of EOAD and other forms of neurodegeneration, not only to gain a more complete understanding of the pathophysiology of EOAD, but also so that targeted therapeutics can be developed to have efficacy in the greatest number of people.
